# Weak antilocalization, spin–orbit interaction, and phase coherence length of a Dirac semimetal Bi_0.97_Sb_0.03_

**DOI:** 10.1038/s41598-022-06776-6

**Published:** 2022-02-21

**Authors:** Yusuff Adeyemi Salawu, Jae Hyun Yun, Jong-Soo Rhyee, Minoru Sasaki, Heon-Jung Kim

**Affiliations:** 1 Department of Physics, Graduate School, Daegu University, Gyeongsan, Gyeongbuk 38453 Republic of Korea; 2 Department of Applied Physics and Institute of Natural Sciences, Kyung Hee University, Yong-In, 17104 Republic of Korea; 3 Department of Physics, Faculty of Science, Yamagata University, Kojirakawa, Yamagata, 990-8560 Japan; 4 Department of Materials-Energy Science and Engineering, College of Engineering, Daegu University, Gyeongsan, Gyeongbuk 38453 Republic of Korea

**Keywords:** Condensed-matter physics, Materials for devices, Applied physics, Condensed-matter physics

## Abstract

The present study develops a general framework for weak antilocalization (WAL) in a three-dimensional (3D) system, which can be applied for a consistent description of longitudinal resistivity $\rho_{xx} \left( B \right)$ and Hall resistivity $\rho_{xy} \left( B \right)$ over a wide temperature (*T*) range. Compared to the previous approach Vu et al. (Phys Rev B 100:125162, 2019), which assumes infinite phase coherence length (*l*_*ϕ*_) and a zero spin–orbit scattering length (*l*_SO_), the present framework is more general, covering high *T* and the intermediate spin–orbit coupling strength. Based on the new approach, the $\rho_{xx} \left( B \right)$ and $\rho_{xy} \left( B \right)$ of the Dirac semimetal Bi_0.97_Sb_0.03_ was analyzed over a wide *T* range from 1.7 to 300 K. The present framework not only explains the main features of the experimental data but also enables one to estimate *l*_*ϕ*_ and *l*_SO_ at different temperatures. The *l*_*ϕ*_ has a power-law *T* dependence at high *T* and saturates at low *T*. In contrast, the *l*_SO_ shows negligible *T* dependence. Because of the different *T* dependence, a crossover occurs from the *l*_SO_-dominant low-*T* to the *l*_*ϕ*_-dominant high-*T* regions. Accordingly, the hallmark features of weak antilocalization (WAL) in $\rho_{xx} \left( B \right)$ and $\rho_{xy} \left( B \right)$ are gradually suppressed across the crossover with increasing *T*. The present theory describes both low-*T* and high-*T* regions successfully, which is impossible in the previous approximate approach. This work offers insights for understanding quantum electrical transport phenomena and their underlying physics, particularly when multiple WAL length scales are competing.

## Introduction

The quantum interference effects of electron waves in a system with linear dispersion have been of great interest in modern condensed matter physics. Recently, several device applications have been proposed that rely on the interference effect in 3D systems^1–3^. The traditionally, well-known technique for detecting this effect has been the measurement of the spin coherence and phase coherence length of the electron wave function. In disordered materials, weak localization (WL) arises from constructive interference between time-reversed partial waves of the charge carriers. This leads to an enhanced probability of carrier backscattering, enhancing resistivity^4–7^. This interference effect is relevant for diffusive orbits up to the length scale of the phase-coherence length *l*_*ϕ*_. At the same time, the spin dynamics of the carriers, which in systems with strong spin–orbit interaction (SOI) is coupled to their orbital motion, introduce interference of time-reversed paths with consequences beyond the WL effect. As the spin experiences a sequence of scattering events along its path, the spin orientation is randomized on a characteristic length scale of spin–orbit scattering length *l*_SO_. The stronger the SOI is, the smaller the *l*_SO_^4,5,8^_._ Here, the interference of time-reversed paths reduces the backscattering probability below its classical value at zero magnetic field. This is the weak antilocalization (WAL) effect, readily observable when *l*_SO_ ≪ *l*_*ϕ*_.

An external magnetic field destroys interference conditions, resulting in a magnetoresistance carrying quantitative information about phase coherence length and spin scattering length^4^. Even though there are several tools to investigate the influence of quantum interference, magnetotransport measurement has become a very effective method to experimentally study this phenomenon. While the influence of quantum interference effects on $\rho_{xx} \left( B \right)$ is well investigated and understood, that on $\rho_{xy} \left( B \right)$ is rarely studied^9^. Because of this, we examined the role of $\rho_{xy} \left( B \right)$ on quantum interference phenomena by using a general framework for weak antilocalization in three dimensions (3D), which applies to the consistent description of both $\rho_{xx} \left( B \right)$ and $\rho_{xy} \left( B \right)$ over the wide temperature ranges.

At low temperatures, the magnetoconductivity with quantum correction $\Delta \sigma$ of two-dimensional (2D) systems is modeled by the Hikami–Larkin–Nagaoka (HLN) theory^8,10,11^. In this theory, four fundamental length scales which determine the *B* dependence of $\Delta \sigma$ exist; the mean free path *l*, which measures the average distance that an electron travels before its momentum is changed by elastic scattering, the phase coherence length *l*_*ϕ*_, which denotes the average distance over which an electron can maintain its phase coherence, the magnetic length *l*_*B*_, and the spin–orbit scattering length *l*_SO_, which represent the strength of the magnetic field and spin–orbit coupling, respectively. These four length scales are necessary to describe the quantum interference effect quantitatively. The phase coherence is usually destroyed by inelastic scattering between electron and phonon or among electrons. To apply the quantum interference effect to experimental data, however, a limiting case is usually taken because the full formula may not exist or be too complex. For instance, the quantum diffusive regime and strong SOI are two widely used limiting cases. In the quantum diffusive regime, where *l*_*ϕ*_/*l* >  > 1, electrons conserve the phase coherence even after being scattered many times. With strong SOI, i.e. *l*_SO_/*l* < 1, the electron wavefunction acquires an additional π phase without losing phase coherence after the electron adiabatically completes a closed trajectory, because of strong coupling between the spin and orbital parts of the wavefunction. This additional π phase is the origin of WAL in a system with strong SOI.

One of the interesting issues in the WL and WAL is the influence of the quantum interference on Hall resistivity $\rho_{xy}$. In a previous study^9^, a method to consistently describe the *B* dependence of $\rho_{xx}$ and $\rho_{xy}$ within the quantum interference framework was suggested. However, this approach deals with a limiting case of infinite *l*_*ϕ*_ and zero *l*_SO_. Because of this limit, the previous method has limited applications, that is, only at low temperatures and for strong SOI cases. Therefore, to extract the temperature dependence of *l*_*ϕ*_ and *l*_SO_ and to fully understand the underlying physics, one needs a theory that properly considers the finite *l*_*ϕ*_ and *l*_SO_. In this study, we extend the previous two-band model with WAL correction by incorporating the effects of finite *l*_*ϕ*_ and *l*_SO_. This naturally allows for quantifying the strength of SOI by estimating the value of *l*_SO_ and provides clues to distinguish different transport regions based on extracted length scales.

The quantum interference effects have been less studied in three-dimensional (3D) systems compared to 2D ones^4,5,8,12–15^ because these effects are weaker in 3D systems. Thus, for a systematic investigation, it is essential to uncover a 3D material that exhibits pronounced quantum interference effects. It is well known that a material with strong SOI has great potential for such a study because strong SOI allows the quantum interference effects to be easily observable.

To examine the detailed effects of *l*_*ϕ*_ and *l*_SO_ on transport properties here, the Bi_1−*x*_Sb*x* (*x* ~ 3–4%) single crystal was chosen. It is a suitable system among the recently identified candidates for topological semimetals; it not only has both electrons and holes carriers, but it also possesses strong SOI. When the concentration of antimony is around *x* ~ 3–4%, massless Dirac fermions emerge near the *L* point in the reciprocal space. This critical concentration is a topological phase transition from a “band” to a topological insulator, also identified as a Dirac semimetal. Accordingly, negative longitudinal magnetoresistance and violation of Ohm’s law were observed, resulting from the chiral anomaly^16–21^. In this study, we would like to systematically explore the quantum interference effects of a Bi_0.97_Sb_0.03_ single crystal over a wide temperature range, offering clues to the relation between band topology and quantum interference effects.

We measured $\rho_{xx} \left( B \right)$ and $\rho_{xy} \left( B \right)$ of the Bi_0.97_Sb_0.03_ single crystal at different temperatures for − 9 T ≤ *B* ≤ 9 T. At low *B* and *T*, we observed a sharp dip or a concave-downward increase in $\rho_{xx} \left( B \right)$, which is a clear manifestation of WAL. This dip, which is distinguished from the conventional *B*-quadratic behavior of $\rho_{xx} \left( B \right)$, becomes suppressed when the temperature is raised. At the same time, $\rho_{xy} \left( B \right)$ is nonlinear in *B* with an S-shape, which is attributed to the combined effects of two bands and the WAL correction. To quantitatively analyze $\rho_{xx} \left( B \right)$ and $\rho_{xy} \left( B \right)$ over wide temperature ranges, we formulated a modified two-carrier model that incorporates all the fundamental length scales of the quantum interference effect. The main features of the experimental data are successfully described by this model for the wide temperature ranges. Also, the key parameters of the system, such as the carrier density, mobility, phase coherence length, etc. are estimated and compared with the values estimated from Shubnikov-de Haas (SdH) oscillations and the previous approximate two-band model, particularly at low temperatures.

## Experiments, results, and discussion

We grew Bi_1−*x*_Sb_*x*_ single crystals at *x* ~ 3–4% using a Bridgeman method, as previously reported^22^. Energy dispersive X-ray analysis was used to measure the concentrations of antimony. The temperature dependences of resistivity $\rho \left( T \right)$ for the Bi_0.97_Sb_0.03_ single crystals were measured from 1.7 to 300 K using a six-probe method. The $\rho_{xx} \left( B \right)$ and $\rho_{xy} \left( B \right)$ measurements were carried out using a cryogen-free magnet system (Cryogenic Inc.) under a magnetic field *B* ranging from − 9 to 9 T. The *B* was applied along the trigonal axis with the current perpendicular to *B* [binary direction (*B* ⊥ *I*)].

A. **The electrical resistivity, magnetoresistance, and Hall resistivity**

**Figure 1:**
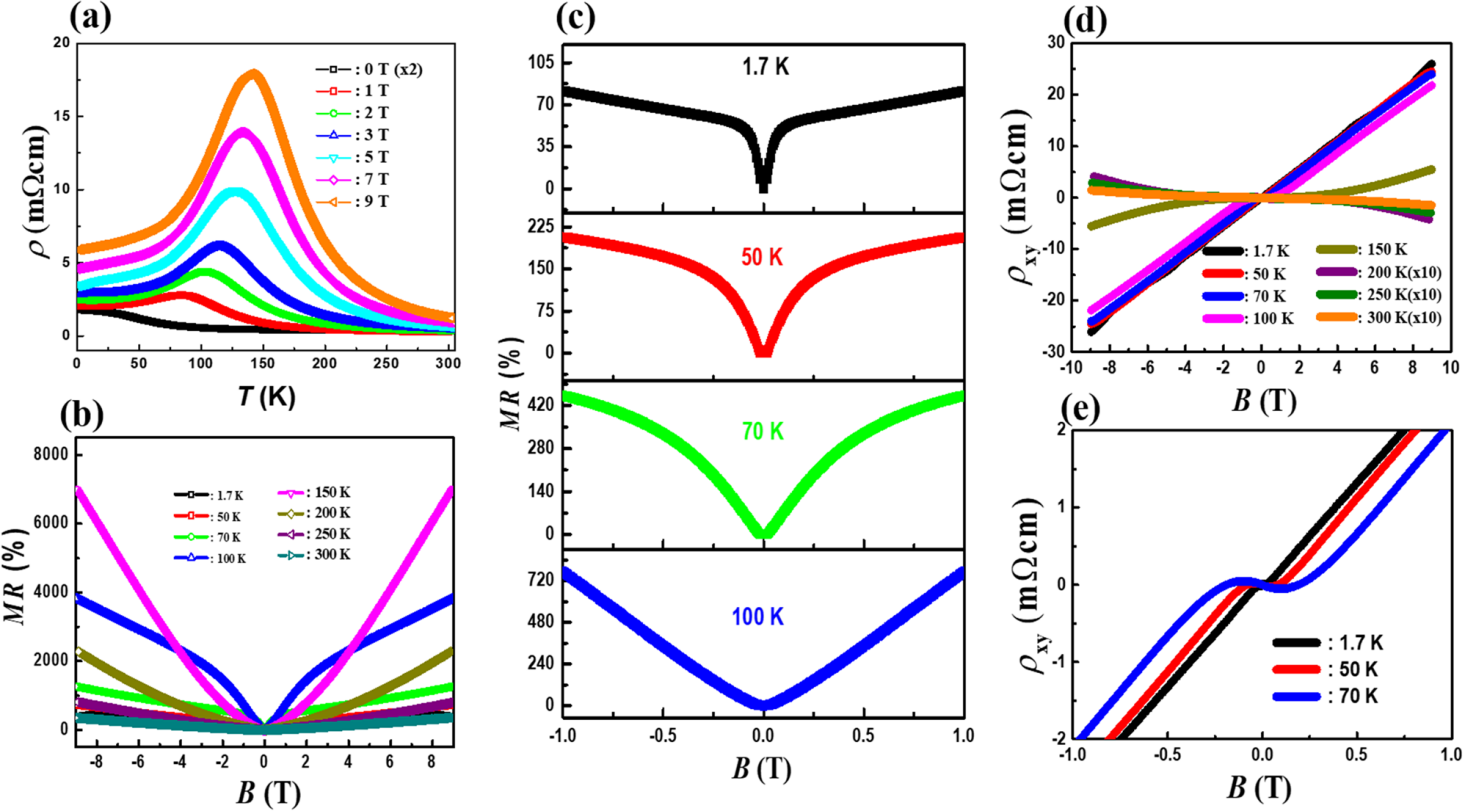
(**a**) Temperature dependences of resistivity *ρ* in a magnetic field perpendicular to the electric current for the Bi_0.97_Sb_0.03_ single crystal. (**b**) Magnetic field dependence of transverse resistivity $\rho_{xx} \left( B \right)$ of the Bi_0.97_Sb_0.03_ single crystal at different temperatures. (c) Enlarge image of transverse resistivity data in low magnetic field at different temperatures (**d**) Hall resistivity $\rho_{xy} \left( B \right)$ of the Bi_0.97_Sb_0.03_ single crystal at various temperatures from 1.7 to 300 K (**e**) Hall resistivity $\rho_{xy} \left( B \right)$ of the Bi_0.97_Sb_0.03_ single crystal at *T* = 1.7 K, 50 K, and 70 K.

Figure 1a displays the temperature dependence of resistivity $\rho \left( T \right)$ curves for a Bi_0.97_Sb_0.03_ single crystal both in the absence and presence of external *B.* The zero-field resistivity of the Bi_0.97_Sb_0.03_ sample exhibits a weak semiconducting behavior below *T* ~ 100 K. Applying *B* perpendicular to the current direction dramatically changes the $\rho \left( T \right)$ curve. Upon the application of *B*, a pronounced peak is developed at *T*_m_ when *B* > 1 T. Here *T*_m_ is the temperature at which the peak occurs. The peak becomes larger with increasing *B*. *T*_m_ appears to signify an “insulator-to-metal” transition. It shifts to higher temperatures as *B* increases. Here, a negative slope $d\rho /dT < 0$ at high temperatures corresponds to an insulating-like region and a positive slope $d\rho /dT > 0$ at low temperatures is a metallic-like region. Such behavior is driven by *B*, which is consistent with previous literature^18^. Possibly, this observation suggests a certain role of Landau level formation particularly at low *T*, but the origin of this transition-like behavior is still elusive.

The result of transverse magnetoresistance *MR* of Bi_0.97_Sb_0.03_ as a function of *B* at different temperatures is shown in Fig. 1b. Here, MR is defined as $MR{ = }\Delta \rho_{xx} {/}\rho_{xx} { = }\left( {\rho_{xx} \left( B \right) - \rho_{xx} \left( 0 \right)} \right)/\rho_{xx} \left( 0 \right) \times 100(\% )$, where $\rho_{xx} \left( B \right)$ and $\rho_{xx} \left( 0 \right)$ are the resistivity values at *B* = *B* and 0 T, respectively. Unexpectedly, MR reaches almost 7000% at *B* = 9 T and *T* = 150 K. Conventionally, the extremely large MR in a semimetal has been attributed to the almost perfect compensation of the hole and electron carriers^22,23^. However, the analysis based on the two-band model with the WAL correction verifies several orders-of-magnitude differences in electron and hole carrier densities, pointing to the fact that a simple compensation scenario is not adequate here. As previously reported, it has already been proposed that the magnetic field in a Dirac semimetal will induce the breaking of the time reversal symmetry, rearranging the Dirac Fermi surface (FS)^17^. This scenario with the high mobility of the Dirac carriers could give rise to extreme MR and is believed to be responsible for the effect observed in our material.

Another notable feature is the *B* dependence of *MR*. According to the semi-classical theory^22,24,25^, the *MR* should increase due to the orbital motion of charge carriers, following $MR \approx \mu_{h} \mu_{e} B^{2}$. However, the experimental *MR*s deviate from this behavior. As shown in Fig. 1b,c, we observed a complete absence of quadratic dependence of $\rho_{xx} \left( B \right)$ at very low *B* and low temperatures. Instead, a sharp dip or concave-downward increase of $\rho_{xx} \left( B \right)$ near the zero-field is present, which is a hallmark of WAL. The presence of the dip strongly implies that the *B* dependence of $\rho_{xx} \left( B \right)$ is not determined solely by the conventional orbital motion of charge carriers. This sharp dip is the strongest at low temperatures, gradually turning into parabolic shapes at higher temperatures.

Figure 1d shows the *B* dependence of Hall resistivity $\rho_{xy} \left( B \right)$ at different temperatures. At low *T*, the $\rho_{xy} \left( B \right)$ curves of the Bi_0.97_Sb_0.03_ single crystal possess a positive slope at high *B*, which indicates the existence of hole carriers in the sample. However, $\rho_{xy} \left( B \right)$ becomes highly nonlinear, particularly at low *B,* as presented in Fig. 1e. This nonlinear $\rho_{xy} \left( B \right)$ at low *B* implies the contribution of the electron carriers, which supports the previous study that reported Bi_0.97_Sb_0.03_ is a two-band system^9^. Interestingly, with increasing temperature, the characteristic *B* at which the slope of the $\rho_{xy} \left( B \right)$ curve turns positive moves to a higher *B*. Eventually, at sufficiently high temperatures, the $\rho_{xy} \left( B \right)$ curve has an overall negative slope. This evolution suggests that certain transport parameters change with temperatures. It is noted that in Fig. 1a, we observed the “insulator-to-metal” transition at about *T* = 150 K and *B* = 9 T. At this temperature, the nonlinearity in $\rho_{xy} \left( B \right)$ is most pronounced, extending to a high *B*.

B. **A new modified two-band model**

The experimental $\rho_{xx} \left( B \right)$ and $\rho_{xy} \left( B \right)$ data are known to be well described by the so-called two-band model when electron and hole carriers coexist in a system^22,26,27^. However, this model does not include quantum interference effects such as WAL or WL. Due to the presence of characteristic features that can be ascribed to WAL in the present experimental $\rho_{xx} \left( B \right)$ and $\rho_{xy} \left( B \right)$ curves of Bi_0.97_Sb_0.03_ single crystals, we developed a new modified two-band model that takes the WAL effects into account. Since we want to explain all features from low to high temperatures, we need a more general theory that does not explicitly assume the limiting values of *l*_*ϕ*_ and *l*_SO_. In natural units $\left( {\hbar = 1{\text{ and }}c = 1} \right)$, $\rho_{xx} \left( B \right)$ and $\rho_{xy} \left( B \right)$ in the conventional two-band theory is given by

1 $$\rho_{xx} \left( B \right) = \frac{{\sigma_{xx} \left( B \right)}}{{\sigma_{xx} \left( B \right)^{2} + \sigma_{xy} \left( B \right)^{2} }},$$

2 $$\rho_{xy} \left( B \right) = - \frac{{\sigma_{xy} \left( B \right)}}{{\sigma_{xx} \left( B \right)^{2} + \sigma_{xy} \left( B \right)^{2} }},$$

3 $$\sigma_{xx} \left( B \right) = \frac{{n_{e} e\mu_{e} }}{{1 + \mu_{e}^{2} B^{2} }} + \frac{{n_{h} e\mu_{h} }}{{1 + \mu_{h}^{2} B^{2} }} = \frac{{n_{e} e^{2} D_{e} }}{{1 + e^{2} D_{e}^{2} B^{2} }} + \frac{{n_{h} e^{2} D_{h} }}{{1 + e^{2} D_{h}^{2} B^{2} }},\;{\text{and}}$$

4 $$\sigma_{xy} \left( B \right) = \left( {\frac{{n_{e} e\mu_{e}^{2} }}{{1 + \mu_{e}^{2} B^{2} }} - \frac{{n_{h} e\mu_{h}^{2} }}{{1 + \mu_{h}^{2} B^{2} }}} \right)B = \left( {\frac{{n_{e} e^{3} D_{e}^{2} }}{{1 + e^{2} D_{e}^{2} B^{2} }} - \frac{{n_{h} e^{3} D_{h}^{2} }}{{1 + e^{2} D_{h}^{2} B^{2} }}} \right)B,$$

here $\sigma_{xx}$ and $\sigma_{xy}$ are magneto-conductivity and Hall conductivity, respectively, and $\mu_{e(h)}$, $n_{e(h)}$, and $D_{e(h)}$ are the mobility, carrier density, and diffusion coefficient of electron (hole), respectively. The relation between mobility $\mu_{e(h)}$ and the diffusion coefficient $D_{e(h)}$ is given by the equation $\mu_{e(h)} = eD_{e(h)}$. In the conventional two-band model, the diffusion coefficient is determined solely by the elastic scattering rate because a single scattering event is dominant. According to the Drude model, the Drude conductivity $\sigma_{D}$ is expressed as $\sigma_{D} = n_{e\left( h \right)} e^{2} D_{e\left( h \right)}$, depending on the type of the charge carrier.

The quantum interference effect modifies the diffusion coefficient. In the present case, we assume the modification of the diffusion coefficient of the electron-doped *L* band as in the previous study^9^. The previous study limited the analysis to the low-temperature region, *i.e.* in the limit of a very long phase-coherence length $\left( {l_{\phi } \to \infty } \right)$ and strong SOI $\left( {l_{SO} \to 0} \right)$. However, in the present case, we need to consider finite phase-coherence length because it is reduced at high temperatures by electron–phonon or electron–electron interactions^1,4^. Therefore, for a quantitative estimation of the WAL effects, we should rely on the full HLN quantum interference theory in 3D^3,5,11^, which fully incorporates four fundamental length scales in the formula. In this framework, the WAL part of conductivity is given by

5 $$\Delta \sigma = ne^{2} D_{e} - ne^{2} D_{0} = N\frac{1}{{8\pi^{2} n\hbar l_{B} }}\left[ {2\zeta \left( {\frac{1}{2},\frac{1}{2} + \frac{{l_{B}^{2} }}{{l^{2} }}} \right) + \zeta \left( {\frac{1}{2},\frac{1}{2} + \frac{{l_{B}^{2} }}{{l_{\phi }^{2} }}} \right) - 3\zeta \left( {\frac{1}{2},\frac{1}{2} + 4 \frac{{l_{B}^{2} }}{{l_{SO}^{2} }} + \frac{{l_{B}^{2} }}{{l_{\phi }^{2} }}} \right)} \right]$$

here *N* is the number of independent interference channels, $D_{0}$ is the diffusion coefficient of the classical diffusive motion ($D_{0} \propto B^{2}$), and $\zeta$ is the Hurwitz zeta function, which has the following two asymptotic forms;

$$\zeta \left( {\frac{1}{2},\frac{1}{2} + x^{2} } \right) \approx \left\{ \begin{gathered} - C_{1} - C_{2} x^{2} ,x \ll 1 \hfill \\ - 2x - 1/48x^{3} ,x \gg 1 \hfill \\ \end{gathered} \right. \quad with\quad \begin{gathered} C_{1} = (1 - \sqrt {2)} \zeta (1/2) \approx 0.605 \hfill \\ C_{2} = (1/2 - \sqrt {2)} \zeta (3/2) \approx 2.39 \hfill \\ \end{gathered}$$

Equation (5) links the WAL part of conductivity with the diffusion coefficients. When the WAL effects are predominant, Eq. (5) can be used, and inserted into Eqs. (3) and (4). In this extended framework, all of the length scales, including the magnetic length $l_{B} = \sqrt {\hbar /4eB}$, the mean free path $l_{{}}$, the phase coherence length $l_{\Phi }$, and the spin–orbit scattering length $l_{SO}$ are properly dealt with. Equation (5) is an extension of the previous formalism, in which a very long phase-coherence length $\left( {l_{\phi } \to \infty } \right)$ and strong SOI $\left( {l_{SO} \to 0} \right)$ were assumed. Because of these approximations, the previous formula cannot be applied to high temperatures and a system with intermediate SOI. We can now use Eqs. (1–4), along with the diffusion coefficient [Eq. (5)] to analyze the $\rho_{xx} \left( B \right)$ and $\rho_{xy} \left( B \right)$ data of the Bi_0.97_Sb_0.03_ single crystal over the entire temperature range from 1.7 to 300 K.

C. **Shubnikov-de Haas (SdH) oscillations and Fermi surface parameters**

**Figure 2:**
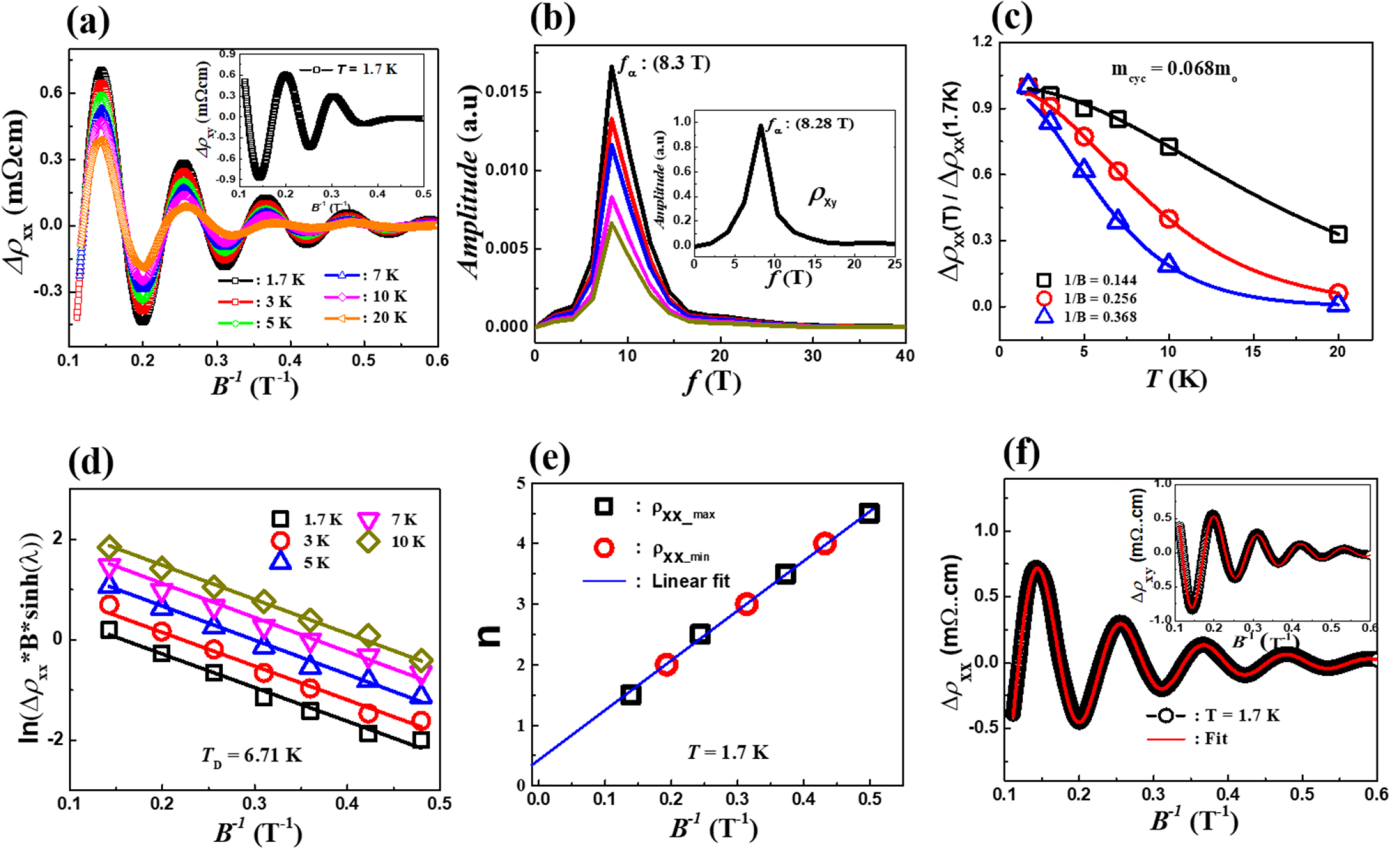
(**a**) Shubnikov-de Haas (SdH) oscillations in the transverse magnetoresistance of a Bi_0.97_Sb_0.03_ single crystal at different temperatures. Insets of (**a**) show the SdH oscillations in the Hall resistivity of a Bi_0.97_Sb_0.03_ single crystal at *T* = 1.7 K. (**b**) Fast Fourier transform (FFT) of the SdH oscillations in $\Delta \rho_{xx} \left( B \right)$ at different temperatures shows a single oscillation frequency. Inset of (**b**) shows FFT of the SdH oscillations in $\Delta \rho_{xy} \left( B \right)$ at *T* = 1.7 K. (**c**) The amplitudes of the SdH oscillations are shown as a function of temperature. A comparison is made between these amplitudes and the Lifshitz-Kosevich equation. (**d**) The amplitude of SdH oscillation versus the inverse magnetic field is plotted to estimate the Dingle equation. (**e**) A Landau fan diagram is constructed from the maximum and minimum of the SdH oscillations. (**f**) The direct fitting of SdH oscillations from the *MR* data to the Lifshits-Kosevich formula. The inset of (**f**) shows the direct fitting of SdH oscillations from the Hall resistivity to the Lifshits-Kosevich formula.

Another interesting feature of electrical transport in Bi_0.97_Sb_0.03_ is the very pronounced SdH oscillations. The presence of the SdH oscillations implies that the Landau level (LL) passes through the Fermi level sequentially with increasing *B*. In Fig. 1b, we trace the SdH oscillations for Bi_0.97_Sb_0.03_ at *B* larger than 2 T. These are the data observed at 1.7 K and show a damping with increasing temperatures. The SdH oscillations are also detected in the Hall resistivity, even though its amplitude is much smaller compared to the transverse MR, as presented in Fig. 1c. The oscillating components of the transverse magnetoresistivity $\Delta \rho_{xx} \left( B \right)$ and Hall resistivity $\Delta \rho_{xy} \left( B \right)$ are extracted from the measured $\rho_{xx} \left( B \right)$ and $\rho_{xy} \left( B \right)$, respectively, by subtracting a suitable background curve. The SdH oscillation has periodic peaks and valleys as a function of the inverse magnetic field *B*^-1^, which indicates the existence of a well-defined Fermi surface and high carrier mobility. Figure 2a shows $\Delta \rho_{xx}$ as a function of inverse magnetic field *B*^-1^ at various temperatures. In the inset in Fig. 2a, we also show $\Delta \rho_{xy}$ at *T* = 1.7 K.

To extract the main frequencies of the SdH oscillations ($\Delta \rho_{xx}$ and $\Delta \rho_{xy}$), a fast Fourier transformation (FFT) analysis was carried out. Here we detected only one oscillation frequency for the $\Delta \rho_{xx}$ and $\Delta \rho_{xy}$ as shown in Fig. 2b and its inset, respectively. This implies that only a single extremal orbit from a single Fermi pocket exists. We observe that the estimated value of frequency for $\Delta \rho_{xx}$ (*f*_α_ = 8.30 T) is almost the same as that of $\Delta \rho_{xy}$ (*f*_α_ = 8.28 T). The extremal cross-sectional area *S*_F_ of the Fermi surface (FS) and the Fermi wave vector are evaluated using the Lifshitz-Onsager relation $S_{{\text{F}}} = \frac{2\pi e}{\hbar }F$ and the formulae $k_{{\text{F}}} = \sqrt {\left( {\frac{{S_{{\text{F}}} }}{\pi }} \right)}$, respectively. The estimated values of $S_{F}$ and $k_{F}$ are 8.65 × 10^−4^ Å^−2^ and 0.0166 Å^−1^, respectively. These values are in reasonably good agreement with the previous report^21^. To estimate the approximate value of carrier density (*n*_SdH_), we further used $S_{F}$ to calculate it by assuming a spherical Fermi surface. The carrier density lies in the range of ~ 1.54 × 10^23^ m^−3^ which is almost the same as the value of the hole carrier density estimated using the new modified two-band model.

The nature of the charge carriers participating in the SdH oscillations can be understood by further analyzing the SdH oscillations based on the Lifshitz-Kosevich (L-K) formula^28–31^, which is expressed by

6 $$\frac{{\Delta \rho_{xx} }}{{\rho_{0} }} \approx \frac{5}{3}\mathop \sum \nolimits_{r = 1} \sqrt {\frac{B}{{2rF_{r} }}} R_{T} \left( {T,r} \right)R_{D} \left( {T_{D} ,r} \right)R_{s} \left( {M_{ZC} ,r} \right)\cos \left[ {2\pi \left( {\frac{{F_{r} }}{B} + \gamma - \delta } \right)} \right]$$

Here, *r* is the orbit index, *F*_*r*_ is the frequency in the orbit *r*, and $\gamma = \frac{1}{2} - \frac{\beta }{2\pi }$ is the Onsager phase factor. This phase originates from the Bohr-Sommerfeld quantization rule for the extremal orbit in the momentum space surrounding the area *S*_*n*_. There are three coefficients in front of the cosine term in Eq. (6), i.e. the thermal damping factor $R_{{\text{T}}} = \frac{{\frac{{2\pi^{2} rk_{{\text{B}}} T}}{{\hbar \omega_{{\text{c}}} }}}}{{\sinh \left( {\frac{{2\pi^{2} rk_{{\text{B}}} T}}{{\hbar \omega_{{\text{c}}} }}} \right)}}$, the Dingle factor $R_{{\text{D}}} = \exp \left( { - \frac{{2\pi^{2} rk_{{\text{B}}} T_{{\text{D}}} }}{{\hbar \omega_{{\text{c}}} }}} \right)$, and the spin factor $R_{{\text{S}}} = \cos \left( {g\frac{{\pi m^{*} }}{{2m_{0} }}} \right)$, where *ω*_c_ is the cyclotron frequency, m^*^ is the cyclotron mass, *T*_D_ is the Dingle temperature, *m*_0_ is the bare mass of an electron, *k*_B_ is the Boltzmann constant, and *ħ* is the Planck constant divided by 2π. The damping by *R*_T_ and *R*_D_ occurs due to Landau level broadening caused by the Fermi–Dirac distribution and the electron scattering, respectively. The spin factor *R*_S_ stems from the Zeeman splitting.

The temperature dependence of the SdH oscillations can be understood based on the thermal damping factor *R*_T_ in the L-K formula. Here, the fit of the temperature dependence of the SdH oscillation amplitude to the thermal damping factor *R*_T_ can give rise to the value of m^*^ as shown in Fig. 2c. We tracked the peak amplitudes of FFT as a function of temperature and these data were compared to the thermal damping factor. For the Bi_0.97_Sb_0.03_ single crystal, the effective mass m^*^ of the charge carriers is approximately 0.067*m*_0_.

The *B* dependence of the SdH oscillation amplitude is described by the Dingle factor *R*_D_^32–38^. Figure 2d displays the so-called Dingle plot for the data at various temperatures. The slope of the Dingle plot is determined by the Dingle temperature *T*_D_, which is 6.7 K for the Bi_0.97_Sb_0.03_ single crystal. Based on the formula $\tau = \frac{\hbar }{{2\pi k_{{\text{B}}} T_{{\text{D}}} }}$ and the Dingle temperature *T*_D_, the scattering time τ was estimated to be *τ* = 1.80 × 10^−13^ s. The mobility was further calculated using $\mu_{{\text{Q}}} = e\hbar /2\pi m_{0} k_{{\text{B}}} T_{{\text{D}}}$. The value of *μ*_Q_ is ~ 0.59 m^2^/Vs, consistent with the values estimated from the MR and Hall fitting based on the new two-band model with the WAL correction.

It is worth noting that the SdH oscillations and low-field transport features cannot be explained by transport through a surface state because surface carrier density is known to be on the order of 10^12^ cm^−2^^33,38,39^, which is much smaller than the estimated values using the SdH oscillations, and magnetoresistance and Hall resistance. Hence, we concluded that the SdH oscillations certainly result from the 3D bulk carriers.

As shown in Fig. 2f, the experimental data for $\Delta \rho_{xx} \left( B \right)$ and $\Delta \rho_{xy} \left( B \right)$ are well fitted to the L-K formula with the parameter values obtained above. These results present more reliable information on the Fermi surface of our system. In particular, the extracted parameter values confirm that the hole carriers dominate (~ 10^23^ m^−3^) in the SdH oscillations at low temperatures.

Next we attempted to estimate phase factors from the SdH oscillations. Under the influence of *B*, a closed orbit is quantized following the Lifshitz-Onsager quantization rule, *i.e.* $S_{n} \frac{\hbar }{eB} = 2\pi \left( {n + \frac{1}{2} - \frac{\beta }{2\pi } - \delta } \right) = 2\pi \left( {n + \gamma - \delta } \right)$, where *β* is Berry’s phase, *δ* is a phase shift that depends on the dimensionality of the system and $\gamma - \delta = \frac{1}{2} - \frac{\beta }{2\pi } - \delta$^32,37,39,40^. The Landau fan (LF) diagram can be constructed using the maxima and minima of the SdH oscillations, which are assigned to the integer (*n*) and half-integer $\left( {n + 1/2} \right)$ indices of the Landau levels, respectively. A linear extrapolation of *n* as a function of 1/*B*_*n*_ leads to the *x*-intercept determined by $\gamma - \delta$ and the slope given by the frequency *F*_*r*_. Thus, the LF diagram is a useful tool for estimating $\gamma - \delta$ and *F*_*r*_. The main source of the error in this estimation is the uncertainty in determining the maxima and minima positions of the oscillations. The system has π Berry’s phase (*β* = π) when the *x*-intercept in the LF diagram is ± 1/8 for a 3D system and 0 for a 2D system. For the 3D case, the sign of *δ* depends on whether the probed extremal cross-sectional area of the FS is maximal ( +) or minimal (−)^35–37,39^. In contrast, a trivial Berry’s phase *β* = 0 is acquired when the *x*-intercept takes − 1/2 ± 1/8 for 3D and − 1/2 for 2D. Here we plot the LF diagram at *T* = 1.7 K. Figure 2e shows the $\gamma - \delta$ value of the Bi_0.97_Sb_0.03_ single crystal, which is estimated to be − 0.1 ± 0.03. The Berry phase of this sample is determined to be 1.45 ± 0.06π (δ = − 1/8) or 0.95 ± 0.06π (δ = 1/8), which is clearly nontrivial.

D. **Analysis and Discussion**

**Figure 3:**
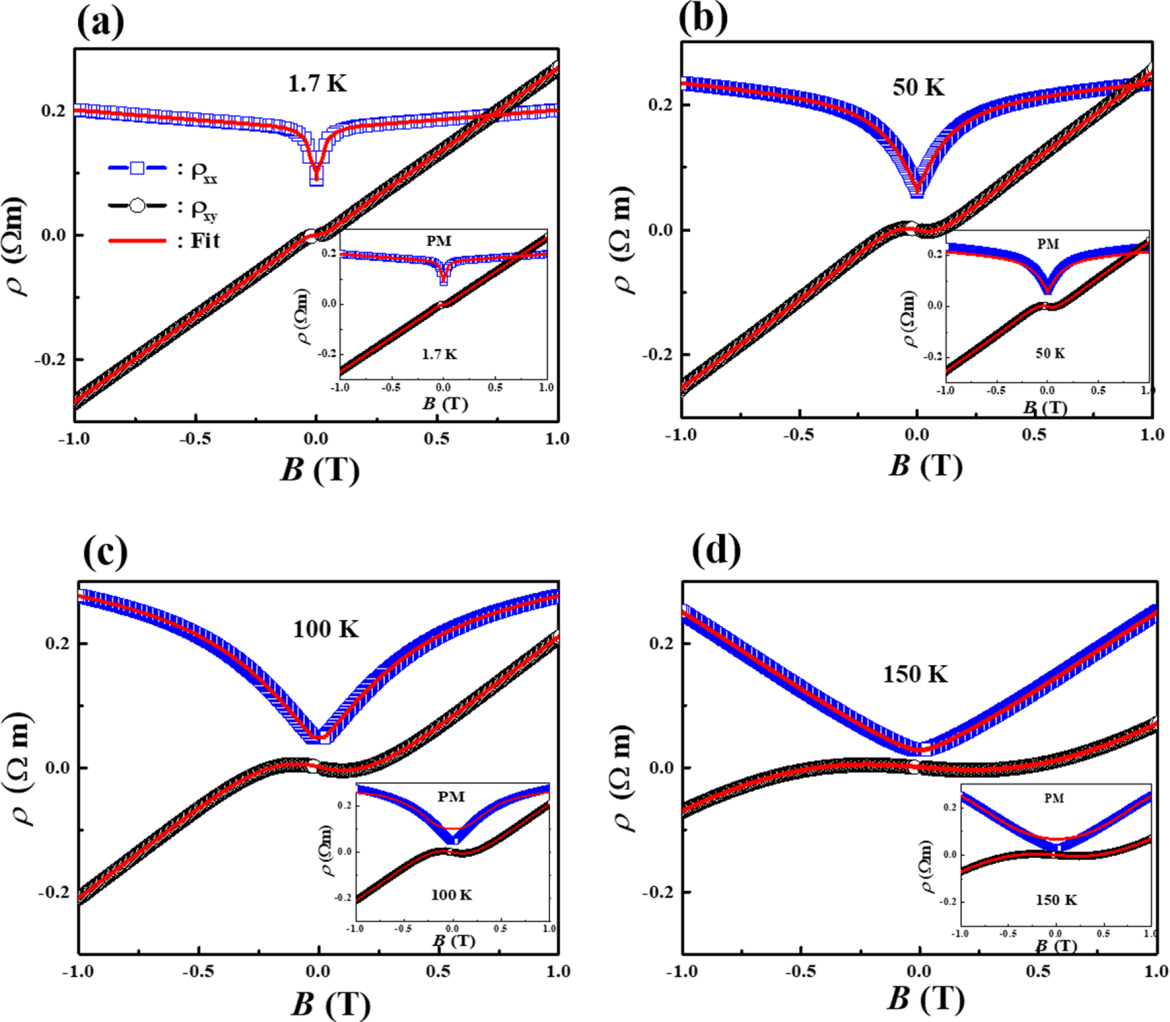
(**a**–**d**) Magnetic field dependence of transverse resistivity $\rho_{xx} \left( B \right)$ and Hall resistivity $\rho_{xy} \left( B \right)$ of the Bi_0.97_Sb_0.03_ single crystal at *T* = 1.7 K, 50 K, 100 K, and 150 K, respectively. The solid lines represent theoretical curves obtained by fitting $\rho_{xx} \left( B \right)$and $\rho_{xx} \left( B \right)$ simultaneously, based on the new modified two-band model. The insets of (**a**–**d**) show theoretical curves obtained by fitting $\rho_{xx} \left( B \right)$and $\rho_{xx} \left( B \right)$ simultaneously, based on the previous model.

In the analysis, we simultaneously fit the $\rho_{xx} \left( B \right)$ and $\rho_{xy} \left( B \right)$ data with one common set of parameters, as shown in Fig. 3. Based on the new modified two-band model with the WAL correction, we successfully fit the experimental data of $\rho_{xx} \left( B \right)$ and $\rho_{xy} \left( B \right)$. We limit the fitting in the range for − 1 T < *B* < 1 T because the model is valid only in the low-field region. Here, the fitting parameters of the analysis are the electron density $n_{e}$, the hole density $n_{h}$, the hole mobility $\mu_{h}$, and the phase coherence length $l_{\Phi }$, the spin–orbit scattering length $l_{{{\text{SO}}}}$, and the mean free path $l_{{}}$ of the electron carriers. At low *T*, the experimental data are excellently simulated by both the new modified two-band theory and the previous model (PM) as shown in Fig. 3a and inset in Fig. 3a, respectively. This demonstrates the reliability of the new method. It is worth noting that by using the new modified two-band model, high-quality fitting for hall resistivity can be easily achieved, not only at low *T* but also at high *T* (See Fig. 3b–d).

**Figure 4:**
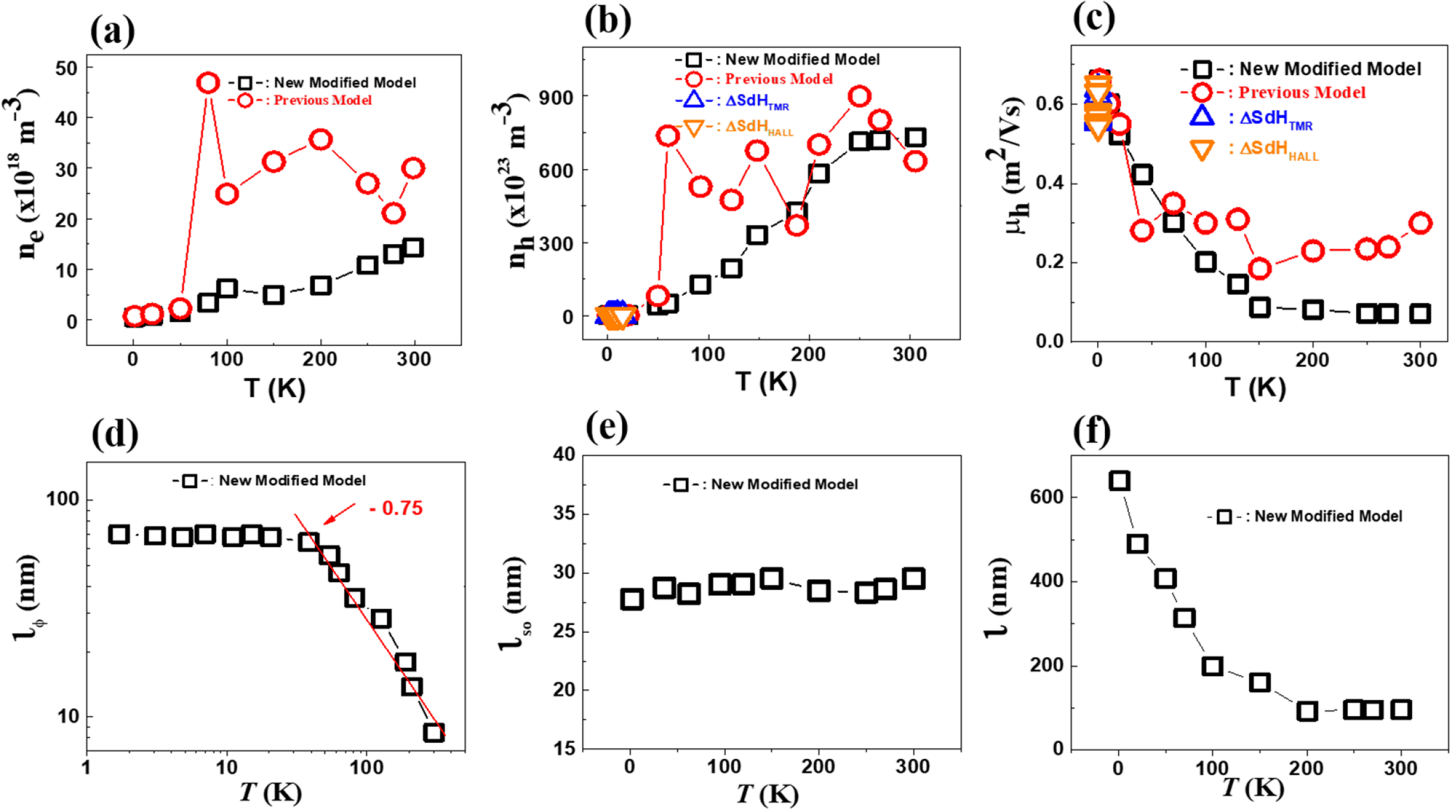
(**a**) The electron density $n_{e}$ (**b**) the hole density $n_{h}$ and (**c**) the hole mobility $\mu_{h}$ as a function of temperatures obtained from the analysis of the new modified two-band model, previous model, and Shubnikov-de Haas (SdH) oscillations. (**d**) the phase coherence length $l_{\Phi }$, (**e**) the spin–orbit scattering length $l_{{{\text{SO}}}}$, and (**f**) the mean free path $l_{{}}$ of the electron carriers as a function of temperatures estimated from the analysis based on the new modified two-band model.

However, the PM fails to reasonably describe the $\rho_{xx} \left( B \right)$ curves at high *T* (see inset in Fig. 3c–d). Moreover, when the PM is utilized, any reasonable parameter values and their *T* trend are not obtained over the wide range of temperatures. This result may be because PM can in principle be applicable only at low temperatures. Since pure Bi is known to have equal numbers of electrons and holes in the range of $n_{e} = n_{h} \sim 10^{23} {\text{ m}}^{ - 3} ,$ the estimated $n_{h}$ value is reasonable. But it is worth noting that $n_{e}$ in the Dirac band is small, indicating that the Fermi energy is close to the Dirac node. The Fermi level of our sample is believed to be closer to the Weyl/Dirac nodes compared to previous reports^21,41^ as revealed by the smaller electron density. In addition, the mobility of the Dirac band in our sample is only 1/100 of the value reported^21,41^. We believe that the present value is more reasonable because it is deduced based on a more complete theory. The electron density $n_{e}$, the hole density $n_{h}$, and the hole mobility $\mu_{h}$ at different temperatures are extracted from fitting, as presented in Fig. 4. In the low-temperature regime, $n_{e}$ and $n_{h}$ obtained in the new modified two-band model are in the range of 10^19^ and 10^23^ m^-3^, as shown in Figs. 4a,b, respectively.

An important feature of $n_{e}$ is its weak temperature dependence below 150 K. On the other hand, $n_{h}$ increases continuously with increasing temperature. At low temperatures, we expected the charge carriers to be trapped in the crystalline defects. However, these trapped charge carriers in the crystalline defects can be thermally excited at higher temperatures, inducing change in the Fermi surface, which causes the increase of both $n_{e}$ and $n_{h}$ with increasing temperature. In addition, we observed that the $\mu_{h}$ increases with decreasing temperature and reaches an extremely high value of $\mu_{h}$≈ 0.63 m^2^/Vs at *T* = 1.7 K, Above 150 K, however, the temperature dependence of $\mu_{h}$ is weaker. At low temperatures, the values of $n_{h}$ and $\mu_{h}$ estimated based on the new modified two-band model are in quantitative agreement with those estimated from the PM and the SdH analysis. This again confirms the reliability of the new modified two-band model for estimating the parameter values.

It is obvious that the present framework not only captures all the essential features of the experimental data but also determines the two primary parameters ($l_{\Phi }$ and $l_{SO}$) that characterize the quantum interference regimes. The values of $l_{\Phi }$ and $l_{SO}$ as a function of temperature extracted from the fitting are shown in Fig. 4d,f, respectively. The $l_{\Phi }$ decreases from 75 to 19 nm monotonically as the temperature increases from 1.7 to 300 K. It is well known that the temperature dependence of $l_{\Phi }$ reflects a dephasing mechanism. Based on theory^1–3^, it follows $l_{\Phi } \propto T^{ - p/2}$ (τ_ϕ_ ∝ T^−*p*^), where *p* depends on the dephasing mechanism. In 3D, *p* = 3/2 and *p* = 3 when the electron–electron and electron–phonon interactions are dominant, respectively. From Fig. 4d, it can be seen that $l_{\Phi }$ increases with decreasing *T* with the exponent *p* = 3/2 at high *T*. This suggests that electron–electron interaction is the main dephasing mechanism at high *T*. The electron–electron interaction is thought to be high because of small carrier density of the electron-doped Dirac band. We also observed saturation of the $l_{\Phi }$ values at low *T* (~ 30 K). Saturation of $l_{\Phi }$ has been widely observed in 2D samples, nanostructures, and 3D systems^1,3,5,7,8^. Here, the $l_{\Phi }$ in our system is believed to be limited by the scattering caused by unscreened Coulomb fluctuations of the charged impurities. These fluctuations are weakened below 30 K, which may be correlated with the saturation of $l_{\Phi }$.

In contrast, the value of $l_{SO}$ was found to be nearly independent of temperature, as shown in Fig. 4d, and it is also noted that the $l_{SO}$ ($l_{SO}$ = 25–29 nm) is the smallest length scale at low temperatures among $l_{\Phi }$, $l_{SO}$, and $l$. This result is understandable because Bi_0.97_Sb_0.03_ is a system with strong SOI. The stronger the SOI is, the smaller *l*_SO_ is. Since $l_{SO}$ is the smallest length scale at low temperatures, the features of the WAL, such as the sharp dip in $\rho_{xx} \left( B \right)$ near zero-field, are stronger at low temperatures. On the other hand, because the condition of *l*_SO_ ≪ *l*_*ϕ*_ does not hold any longer at high temperatures, all the features related to WAL become weakened. Bi_0.97_Sb_0.03_ undergoes a crossover from *l*_SO_-dominant low-*T* to *l*_*ϕ*_-dominant high-*T* regions when the *T* dependences of $l_{\Phi }$ and $l_{SO}$ cross.

In addition, extrinsic spin–orbit scattering, or the spin–orbit disorder can give a nontrivial effect on WL and WAL of massless Dirac fermion systems as reported^42,43^. This effect could tend to suppress the weak localization (WL) channel, extending the region of weak antilocalization (WAL). Therefore, the spin–orbit disorder will act as increasing the WAL tendency and decrease the $l_{SO}$ values in the present case.

The mean free path $l_{{}}$ of the electron band extracted from the fit is plotted in Fig. 4f. The value of $l_{{}}$ decreases from 640 to 90 nm monotonically with increasing *T*. From this, we estimate the electron mobility $\mu_{e}$, which gives $\mu_{e}$ = 17.5–3.7 m^2^/Vs. The value of the electron mobility is larger than that of the hole mobility. $l_{\Phi }$ is one order of magnitude smaller than $l_{{}}$. This indicates that an electron’s phase is randomized before a few elastic collision events occur. The present formula was formulated to explain all the possible diffusive regimes that may present in our system. In this case, it is believed that our system does not reach quantum diffusive regime, but it is close to this region.

**Figure 5:**
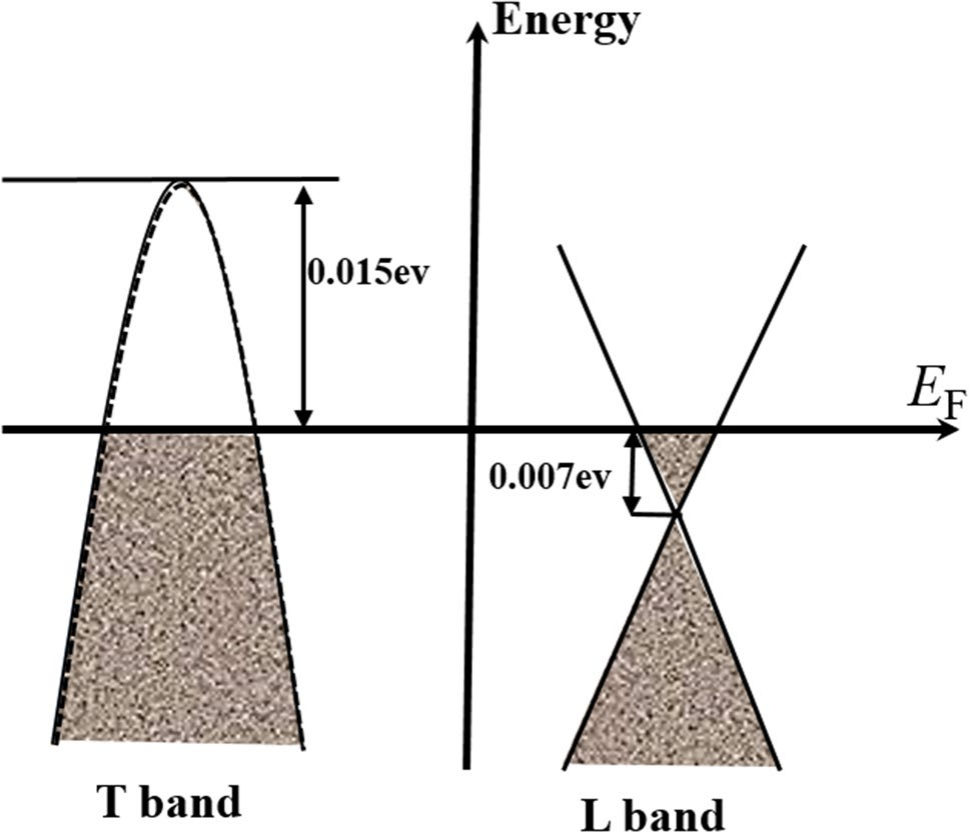
The position of the Fermi level at the *T* and *L* bands deduced from the SdH oscillations analysis, and new modified two-band model fitting.

Finally, we discuss the position of the Fermi level *E*_F_ at the *L* and *T* band as shown in Fig. 5. In this system, the Fermi energy lies between the Dirac node and the top of the valence band as shown in Fig. 5. Because of this, we use both the hole and electron carrier densities to estimate the location of the Fermi level. Based on the relation $n_{e} = \frac{{4/3\pi k_{F}^{3} }}{{{(2}\pi /L)^{3} }} = \frac{1}{{6\pi^{2} }}Vk_{{_{F} }}^{3}$, we can calculate the Fermi vector *k*_F_ of the Dirac band at *L* point. Here, $n_{e}$ was estimated to be 0.54 × 10^18^ m^−3^ from the new modified two-band model. The Fermi energy $E_{{\text{F}}}$ also satisfies the relation, $E_{{\text{F}}} = v\hbar k_{{\text{F}}}$ for the linear band dispersion. Thus $E_{{\text{F}}} = v\hbar k_{{\text{F}}}$ = 0.007 eV with the Fermi velocity of *v* = 10^6^ m/s^21^. We also estimated the Fermi level of the hole band at *T* point from the top of the valence band. In a quadratic band, we have the following formula: $n_{{\text{p}}} = 2\frac{{4/3\pi k_{F}^{3} }}{{{(2}\pi /L)^{3} }} = \frac{1}{{2\pi^{2} }}Vk_{{_{F} }}^{3}$ and $E_{{\text{F}}} = \frac{{\hbar^{2} {\text{k}}^{2} }}{{{\text{2m}}^{*} }}$. Using the effective mass of m* = 0.068m_e_ estimated from the SdH analysis, $E_{{\text{F}}}$ is calculated to be 0.015 eV. The energy difference between the nodal point and the top of the valence T band is 0.022 eV, which is slightly larger than the value in the previous report^21,44^. This means that the Fermi level is closer to the nodal point, and the density of states from the *T* band is larger compared to the previous case^44^. This is very consistent with the fact that the negative longitudinal MR and the violation of Ohm’s law are quite pronounced in the present sample. Overall, our estimated $E_{{\text{F}}}$ position is remarkably consistent with the reported value^21^ and so it proves the validity of the MR, Hall, and SdH analysis. The closeness of $E_{{\text{F}}}$ to the Dirac node is thought to be the main reason for why the topological transport phenomena are easily observed, such as negative longitudinal MR and violation of Ohm’s law in this Bi_0.97_Sb_0.03_ single crystal.

## Conclusion

In this study, we conducted a comprehensive investigation into the magneto-transport properties of a Bi_0.97_Sb_0.03_ single crystal at temperatures up to 300 K and with *B* up to ± 9.0 T. At low *T*, *MR* does not show conventional *B*-quadratic behavior, but a sharp dip appears at low *B* in this system, whose origin is ascribed to weak antilocalization. This sharp dip is the strongest at low temperatures, becoming parabolic shaped at higher temperatures. On the other hand, Hall resistivity is nonlinear in *B* with an S-shape, which is caused by the combined effect of two bands and the WAL correction. To analyze the experimental data beyond low temperatures and to describe the experimental data even at high temperatures, we employed a new approach of including a WAL correction. The experimental data were excellently simulated by these theoretical curves and all the main features that were not captured by the previous modified two-band theory are also described successfully. This strongly suggests the reliability of the new approach and the interplay of WAL and two distinct charge carriers in the Bi_0.97_Sb_0.03_ single crystal. Also, we complement the measurement of these transport quantities by using Shubnikov-de Haas oscillations of the MR and Hall resistivity.
